# The Relationship Between PANC-3 Score and Nutritional Risk Screening 2002 Score in Patients with Acute Pancreatitis

**DOI:** 10.5152/tjg.2026.25506

**Published:** 2026-01-19

**Authors:** Abdurrahman Ercan, Yağmur Kınacı Gümüşçubuk, Nuray Yılmaz Çakmak, Zeki Mesut Yalın Kılıç

**Affiliations:** 1 Department of Internal Medicine, Giresun Training and Research Hospital, Giresun, Türkiye; 2 Department of Occupational Disease, Hacettepe University Faculty of Medicine, Ankara, Türkiye; 3 Department of Palliative Center, Ankara Yıldırım Beyazıt University Faculty of Medicine, Ankara, Türkiye; 4 Department of Gastroenterology, Ankara City Hospital, Ankara, Türkiye

**Keywords:** Acute pancreatitis, NRS-2002, nutrition, PANC-3

## Abstract

**Background/Aims:**

Early identification and severity assessment of acute pancreatitis (AP) are crucial for preventing adverse clinical outcomes. The objective of this study was to assess the efficacy and clinical applicability of the PANC-3 and Nutritional Risk Screening 2002 (NRS-2002) scoring systems in estimating disease severity in patients with AP. This study addresses the limited number of studies assessing the use of NRS-2002 in this specific clinical condition.

**Materials and Methods:**

This prospective observational study was executed between July and October 2023 and included patients who were hospitalized with a diagnosis of AP. A total of 203 patients over the age of 18 were enrolled. Patients with active malignancy, chronic liver disease, or pregnancy were excluded.

**Results:**

Patients with a PANC-3 score of 3 or an NRS-2002 score of 3 or higher experienced significantly more local and/or systemic complications, longer hospital stays, and a greater need for higher-level intensive care compared to those with lower scores (*P* < .001). The NRS-2002 score ≥3 was significantly linked with increased mortality (*P* < .001) and was described as an independent risk factor in multivariate analysis (*P* = .003).

**Conclusion:**

The PANC-3 score provides a practical tool for early prediction of AP severity and may help prevent disease progression with timely intervention. The findings suggest that patients with an NRS-2002 score of 3 or higher are at nutritional risk and tend to have a more severe process of AP. Early assessment of nutritional status and appropriate nutritional support may reduce disease severity in these patients.

**Main Points**

- In this study, Nutritional Risk Screening 2002 (NRS-2002) ≥3 was revealed to be an independent risk factor for mortality.
- Patients with a PANC-3 score of 3 or an NRS-2002 score ≥3 experienced significantly higher rates of local and/or systemic complications compared to those with lower scores.
- Both a PANC-3 score of 3 and an NRS-2002 score ≥3 were shown as independent risk factors for prolonged hospitalization. The PANC-3 score, consisting of 3 simple parameters, can provide early information about the severity of acute pancreatitis (AP).
- Among patients with NRS-2002 ≥3, those identified as nutritionally at risk showed a tendency toward more severe progression of AP.

## Introduction

Acute pancreatitis (AP) is an inflammatory condition of the pancreas that can result from various etiologic factors. The diagnosis of AP requires at least 2 of the following 3 criteria: acute onset of abdominal pain, serum amylase or lipase levels elevated to at least 3 times the upper limit of normal, and radiologic findings showing pancreatic heterogeneity and peripancreatic fat stranding.^1^ Alcohol consumption and bile duct stones are the leading etiological factors of AP. In addition, hypertriglyceridemia, certain medications, genetic predisposition, and autoimmune disorders may contribute to its development.^2^^,^^3^

Approximately 80% of AP cases are mild and usually resolve spontaneously. The severe form, while less common, has a mortality rate approaching 30%. Early diagnosis and treatment of severe pancreatitis are pivotal in reducing mortality and morbidity rates.^4^^,^^5^

Early diagnosis of patients at risk for serious complications of AP remains a clinical challenge. Although severe disease occurs in fewer than 30% of cases, it accounts for more than 99% of AP-related deaths. Therefore, identifying and managing high-risk patients within the first 24 hours is critical to preventing severe outcomes.^6^ Patients with severe AP benefit significantly from early intensive care management. Hence, early assessment of disease severity is vital to guide timely and appropriate treatment decisions.^7^^,^^8^

Various scoring systems have been improved to estimate the severity of AP, each with its own strengths and limitations.^9^ Among them, the PANC-3 scoring system is preferred due to its simplicity, wide availability, and cost-effectiveness.^10^ When applied during hospitalization, it has been demonstrated to estimate disease severity as effectively as the APACHE II score.^11^ The Nutritional Risk Screening 2002 (NRS-2002) score, presented by Kondrup et al^12^ nearly 20 years ago, is a commonly used tool for evaluating nutritional status across various clinical populations and is recommended in several clinical guidelines.^13^ However, studies evaluating the application of NRS-2002 in patients with AP are still limited.

Although numerous studies have been conducted on various parameters and scoring systems, there is currently insufficient evidence or consensus on a “gold standard” prognostic score for predicting severe AP. Because patients with AP are at risk of developing permanent organ failure, it is important to classify the severity of AP early.^4^

This study aims to assess the predictive accuracy of the PANC-3 and NRS-2002 scores in determining disease severity in AP and to investigate whether these scores can be effectively used in this patient group.

## Materials and Methods

The study was performed by the Department of Internal Medicine at Ankara City Hospital. The study was designed in line with the Patient Rights Regulation, the Good Clinical Practice guidelines, and the principles of the Declaration of Helsinki (2013 revision).

The study included patients who sought care at the Emergency Department, were diagnosed with AP, and were subsequently hospitalized and followed by the Departments of Internal Medicine and Gastroenterology. This prospective observational study was conducted between July 20, 2023, and October 10, 2023. All patients who participated in the study provided informed consent. This study was approved by the Ethics Committee of Ankara City Hospital (approval number: E2-23-4348; date: July 23, 2023).

Acute pancreatitis was identified relying on the identification of at least 2 of the following criteria: characteristic belt-like abdominal pain extending to the back, serum amylase and/or lipase levels greater than 3 times the highest limit of normal, and specific radiological findings such as fat stranding around the pancreatic parenchyma.

The PANC-3 score was considered positive if all 3 of the following were present: hematocrit >44%, body mass index (BMI) >30 kg/m^2^, and pleural effusion on chest imaging.^10^

The NRS-2002 scoring system consists of 2 parameters: nutritional status and disease severity and is scored as no problem, mild, moderate, and severe. Each section is scored from 0 to 3. Additionally, an extra point is added for patients aged 70 and over. Patients with a total score of ≥3 are considered to be at nutritional risk, and a nutritional assessment is recommended for these patients.^13^ The NRS-2002 scoring steps are presented in Tables 1 and 2.

The primary outcome of the study was the development of local or systemic complications linked with AP, including acute necrotic collections, peripancreatic or extrapancreatic fluid collections, or walled-off necrosis (WON).

Secondary endpoints included severe clinical outcomes of AP, such as prolonged hospitalization (>10 days), admission to second- or third-level intensive care, or death.

### Statistical Analysis

Statistical analyses were executed using SPSS version 26 (IBM SPSS Corp.; Armonk, NY, USA). Several normality tests were applied to evaluate the distribution of continuous variables, among them the Kolmogorov–Smirnov and Shapiro–Wilk tests. Variables exhibiting a normal distribution were expressed as mean ± standard deviation, whereas those not normally distributed were expressed as median (range). Categorical variables were expressed as counts and percentages (%).

Categorical variables were evaluated using Pearson’s chi-square test and Fisher’s exact test. The Student *t*-test was used for normally distributed continuous variables, and the Mann–Whitney *U*-test was used for non-normally distributed ones.

Variables demonstrated to be statistically significant in univariate analyses were further examined using univariate and multivariate regression analyses. receiver operating characteristic (ROC) analysis was performed to assess the predictive ability of the NRS-2002 and PANC-3 scores for mortality. The area under the curve (AUC) was calculated for each score, and the optimal cut-off values were determined based on the highest sensitivity and specificity balance. A *P* value less than .05 was regarded as statistically significant. In addition, odds ratios were determined with 95% CIs.

## Results

When patients with a PANC-3 score of 3 were compared to those with scores <3, statistically notable differences were observed in the incidence of acute necrotic collection, peripancreatic fluid collection, extrapancreatic fluid collection, and WON. These complications were significantly more frequent among patients with a PANC-3 score of 3. Additionally, prolonged hospitalization (>10 days) and the need for second- or third-level intensive care were significantly higher among patients with a PANC-3 score of 3 (*P* < .001). The relationship between PANC-3 scores, complications, and clinical outcomes is presented in Table 3.

When patients with an NRS-2002 score ≥3 were compared to those with scores <3 in terms of complications, statistically significant differences were revealed in the occurrence of pleural effusion, extrapancreatic fluid collection, and WON (*P* < .001). These complications were more frequently seen in patients with an NRS-2002 score ≥3. The relationship between NRS-2002 scores, complications, and clinical outcomes is presented in Table 4.

When patients who experienced mortality were compared with those who survived, statistically significant associations were found between mortality and the presence of pleural effusion, WON, and an NRS-2002 score ≥3 (*P* < .001). The relationships between mortality, complications, scoring systems, and comorbidities are summarized in Table 5.

In multivariate regression analysis, having an NRS-2002 score ≥3 was identified as an independent risk factor for mortality, with an odds ratio of 14.019 (95% CI: 2.514-78.166; *P* = .003). The regression analysis of factors linked with mortality is presented in Table 6.

Both a PANC-3 score of 3 and an NRS-2002 score ≥3 were found to be significant independent risk factors for prolonged hospital stay in the multivariate regression analysis. The regression analysis of factors associated with prolonged hospitalization is presented in Table 7.

**Figure 1.**
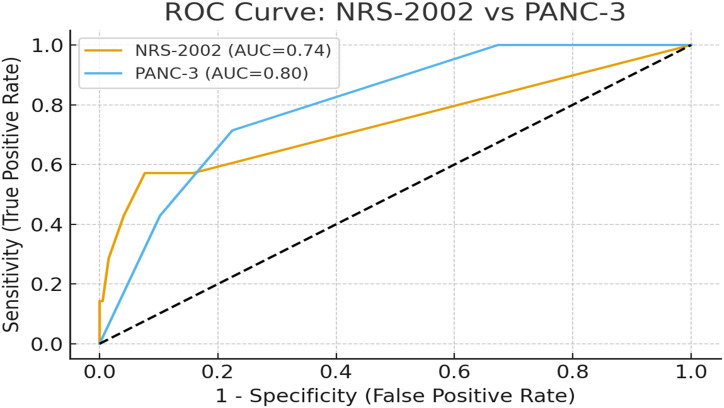
Roc curves for NRS-2002 and PANC-3.

The ROC analysis was performed to evaluate the predictive ability of the NRS-2002 and PANC-3 scores for mortality. For the NRS-2002 score, the area under the ROC curve (AUC) was 0.738 (95% CI: 0.501-0.975; *P* = .033). At the optimal cut-off value of ≥3, the discriminative ability for mortality was calculated with a sensitivity of 57.1% and a specificity of 92.3% (Youden index = 0.495). For the PANC-3 score, the AUC was 0.803 (95% CI: 0.663-0.943; *P* = .006). At the optimal cut-off value of ≥2, the discriminative ability for mortality was observed with a sensitivity of 71.4% and a specificity of 77.6% (Youden index = 0.490). Overall, both scores demonstrated a statistically significant discriminative ability in relation to mortality, with the PANC-3 score showing higher discriminative performance compared to the NRS-2002 score (Figure 1).

## Discussion

Acute pancreatitis is a common condition seen in emergency departments. The AP severity is classified as mild, moderate, and severe according to the presence of local and systemic complications, necrosis, and infected necrosis status. The majority of cases are mild and resolve spontaneously within 3-5 days. In contrast, severe AP occurs in approximately 15%-20% of all cases, and the associated mortality can vary between 10% and 85% depending on the center and country.^14^^,^^15^

There is insufficient evidence and no consensus on a “gold standard” prognostic score to predict severe AP. Severe AP has high mortality and morbidity rates, which require early identification of potential complications for aggressive treatment. Rapid and accurate prediction of severe AP progression is pivotal for improving patient prognosis.^4^ Therefore, there is a need for an early predictor of AP severity that is both sensitive and specific enough to be clinically reliable.^11^^,^^16^ This study aimed to evaluate the usability of the PANC-3 and NRS-2002 scores—both of which include parameters that are easily accessible in all healthcare facilities and are easy to apply—for early prediction in patients diagnosed with AP.

In a previous study, no statistically significant difference was found between the predictive values of APACHE II and PANC-3 scores in determining the severity of AP.^11^ Similarly, a study by Beduschi et al^17^ demonstrated that the PANC-3 score had high specificity and accuracy compared to the Revised Atlanta Classification, with a strong predictive value for severe AP.

Peripancreatic fluid collections that occur in cases of acute interstitial edematous pancreatitis rarely evolve into pseudocysts or become infected, and usually follow a benign clinical course with spontaneous resolution. However, in patients with a diagnosis of acute necrotizing pancreatitis, acute necrotic collections have the potential to become infected, transform into WON, or lead to other complications, resulting in a severe clinical picture and increased mortality and morbidity. Therefore, identifying fluid collections early after the onset of AP is of great importance for guiding treatment and follow-up.^18^^,^^19^ A review of the literature shows that pancreatic collections are more frequently observed in patients diagnosed with severe AP. Similarly, in this study, acute necrotic collection, extraparenchymal fluid collection, peripancreatic fluid collection, and WON were significantly more common in patients with a PANC-3 score of 3. Additionally, pleural effusion, extraparenchymal fluid collection, and WON were found to be significantly higher in patients with an NRS-2002 score of ≥3 (*P* < .001).

In a study by Beduschi et al,^17^ when clinical outcomes were compared based on the PANC-3 score, a positive score was not associated with hospital stay duration or mortality. Yet, it was noted that patients with a positive PANC-3 score more often needed intensive care and experienced prolonged ICU stays. Similarly, in this study, patients with a PANC-3 score of 3 had a significantly greater need for intensive care (*P* < .001). Moreover, 42.8% of the patients who died had a PANC-3 score of 3, and this was found to be statistically significantly linked with mortality (*P* = .007).

In the literature, a few studies have researched the link between the NRS-2002 score and AP. In the study by Chen et al,^20^ the NRS-2002 score and 2 other nutritional assessment scores were evaluated for estimating mortality in patients diagnosed with severe AP. The NRS-2002 score was found to be a statistically significant predictor of 90-day mortality. In the study, mortality was observed in 21.1% of patients with an NRS-2002 score of ≥3, compared to 1.6% in those with a score <3. Among the patients who died, 57.1% had an NRS-2002 score of ≥3, and this association with mortality was statistically significant (*P* < .001).

In a study conducted in Norway, among patients with AP and other pancreatic diseases, those at risk for malnutrition (defined as a score ≥3 in the NRS-2002) had a longer length of hospital stay compared to those without malnutrition risk (*P* = .044). The 1-year mortality rate was higher in patients at risk for malnutrition (16.4%) compared to those not at risk (3.6%). However, after adjusting for factors such as age, sex, BMI, and comorbidities, the relationship between malnutrition risk and survival was not statistically significant.^21^ The findings further confirm and extend these observations, demonstrating stronger associations between NRS-2002 scores and clinical outcomes. Patients with NRS-2002 scores ≥3 had significantly higher rates of prolonged hospitalization (84.2% vs. 35.3%, *P* < .001) and intensive care requirements (63.2% vs. 10.3%, *P* < .001). Notably, the multivariate regression analysis identified NRS-2002 score ≥3 as an independent risk factor for both mortality (OR: 14.019, 95% CI: 2.514-78.166; *P* = .003) and prolonged hospital stay. The ROC analysis revealed that NRS-2002 score had significant discriminative ability for mortality prediction with an AUC of 0.738 (95% CI: 0.501-0.975; *P* = .033), achieving 57.1% sensitivity and 92.3% specificity at the optimal cut-off of ≥3. These results suggest that NRS-2002 scoring may serve as a valuable tool for predicting not only nutritional risk but also overall disease severity in AP patients.

Various studies in the literature showed higher mortality rates in patients who developed local or systemic complications.^22^^,^^23^ Similarly, in the study, pleural effusion, acute necrotic collection, extraparenchymal fluid collection, and WON were associated with higher mortality. Both a PANC-3 score of 3 and an NRS-2002 score of ≥3 were shown as independent risk factors for mortality. Furthermore, both scores were revealed to be independent risk factors for prolonged hospital stay.

A major limitation of the study is that it was conducted at a single center and included only patients hospitalized in the Internal Medicine and Gastroenterology departments. However, since the hospital receives referrals from all regions of the country, the study population represents a broad demographic.

Another limitation is that some of the parameters in the NRS-2002 score rely on patient self-reporting. The details of nutritional support (enteral or parenteral, timing, calorie/protein targets) for patients with high NRS-2002 scores were not included in the study. In the country, patients have rapid access to healthcare and are often diagnosed in the early stages, allowing for timely initiation of treatment. As a result, the number of severe AP cases was relatively low in this study. Additionally, the cross-sectional design of the study is also a limitation.

In this study, patients diagnosed with AP were evaluated using the PANC-3 and NRS-2002 scoring systems. Among patients with a PANC-3 score of 3, local and/or systemic complications, prolonged hospital stays, and the need for level 2 or 3 intensive care were found to be significantly higher.

Based on these findings, the PANC-3 scoring system may enable rapid estimating of disease course in patients presenting with AP due to its simplicity, ease of application, widespread availability, and low cost compared to other systems. Patients with a score of 3 should be closely monitored and considered for early intensive care admission, which may reduce mortality rates.

When patients with AP were evaluated using the NRS-2002 score in the study, those with a score of ≥3 were found to have significantly higher rates of local and/or systemic complications and mortality.

Therefore, patients who are at nutritional risk tend to have a more severe disease course. Providing timely and appropriate enteral or parenteral nutritional support and correcting caloric deficits may help prevent disease progression and reduce mortality.

## Figures and Tables

**Table 1.** NRS-2002 Score—Step 1

**No**	Screening Question	Yes	No
1	Is the BMI below 20.5 kg/m^2^?	□	□
2	Has there been weight loss during the last 3 months?	□	□
3	Has food intake decreased during the last week?	□	□
4	Is there a severe illness present? (e.g., intensive care unit patient)	□	□

Interpretation: If any answer is “Yes” → proceed to Step 2. If all answers are “No” → the patient should be re-screened weekly.

**Table 2.** NRS-2002 Score—Step 2

**Impairment in Nutritional Status**	Score	Severity of Disease	Score
Normal nutritional status, no impairment	0	No increase in nutritional requirements, no disease	0
>5% weight loss in 3 months or food intake during the last week was 50%-75% of normal requirements	1	Mild disease: Hip fracture, chronic diseases with acute complications (cirrhosis, COPD, dialysis, diabetes, cancer)	1
>5% weight loss in 2 months or BMI 18.5-20.5 kg/m^2^ + food intake during the last week was 25%-60% of requirements	2	Moderate disease: Major abdominal surgery, stroke, severe pneumonia, hematologic malignancy	2
>5% weight loss in 1 month (or >15% in 3 months) or BMI <18.5 kg/m^2^ + impaired general condition or food intake <25% of requirements	3	Severe disease: Head trauma, bone marrow transplantation, ICU patients (APACHE II >10)	3

APACHE II, Acute Physiology and Chronic Health Evaluation II; BMI, body mass index; COPD, chronic obstructive pulmonary disease; ICU, intensive care unit.

**Table 3.** The Relationship Between PANC-3 Scores, Complications, and Clinical Outcomes

	PANC-3 Score <3	PANC-3 Score = 3	All	*P*
Extrapancreatic fluid collection	34 (18.9%)	19 (82.6%)	53 (26.1%)	<.001
Peripancreatic fluid collection	45 (25%)	18 (78.3%)	63 (31v)	<.001
Pseudocyst	5 (2.8%)	1 (4.3%)	6 (3%)	
Acute necrotizing collection	9 (5%)	10 (43.5)	19 (9.4%%)	<.001
WON (Walled-off necrosis)	6 (3.3%)	6 (26.1%%)	12 (5.9)	<.001
Need for second or third level intensive care	19 (10.6%)	12 (52.2%)	31 (15.3%)	<.001
Mortality	4 (2.2%)	3 (13%)	7 (3.4%)	.007
Prolonged hospitalization (>10 days)	64 (35.6%)	17 (73.9%)	81 (39.9%)	<.001
Length of hospital stay	9 (1-98)	18 (7-98)	9 (1-98 )	<.001
Intensive care length of stay	0 (0-85)	2 (0-40)	0 (0-85)	<.001

**Table 4.** The Relationship Between NRS-2002, Complications, and Clinical Outcomes

	NRS-2002 Score <3	NRS-2002 Score ≥ 3 n = 19 (9.3%)	*P*
Pleural effusion	32 (17.4%)	10 (52.6%)	<.001
Extrapancreatic fluid collection	42 (22.8%)	11 (57.9%)	.001
Peripancreatic fluid collection	53 (28.8%)	10 (52.6%)	.033
Pseudocyst	6 (3.3%)	0 (0%)	
Acute necrotizing collection	16 (8.7%)	3 (15.8%)	.312
WON (Walled-off necrosis)	6 (3.3%)	6 (31.6%)	<.001
Need for second or third level intensive care	19 (10.3%)	12 (63.2%)	<.001
Mortality	3 (1.6%)	4 (21.1%)	0.001
PANC-3 Score = 3	20 (10.9%)	3 (15.8%)	.519
Prolonged hospitalization (>10 days)	65 (35.3%)	16 (84.2%)	<.001
Length of hospital stay	9 (1-98)	18 (7-98)	<.001
Intensive care length of stay	0 (0-40)	10 (0-85)	<.001

NRS-2002, Nutritional Risk Screening 2002.

**Table 5.** The Relationships Between Mortality, Complications, Scoring Systems, and Comorbidities

	Patients Without Mortality	Patients with Mortality n = 7 (3.4%)	*P*
Pleural effusion	36 (18.4%)	6 (85.7%)	<.001
Extrapancreatic fluid collection	48 (24.5%)	5 (71.4%)	.005
Peripancreatic fluid collection	59 (30.1%)	4 (57.1%)	.129
Acute necrotizing collection	16 (8.2%)	3 (42.9%)	.002
WON	7 (3.6%)	5 (71.4%)	<.001
Congestive heart failure	7 (3.6%%)	2 (28.6%)	.002
PANC-3 Score = 3	19 (9.7)	3 (42.8%)	.007
NRS-2002 Score ≥3	15 (7.6%)	4 (57.1%)	<.001

NRS-2002, Nutritional Risk Screening 2002.

**Table 6.** Regression Analysis Examining Factors Linked to with Mortality

	Univariate		Multivariate*	
	OR (9%5 GA)	Significance	OR (95% GA)	Significance
PANC-3 Score = 3	6.6 (1.378-31.621)	0.018	8.492 (1.559-46.254)	0.013
NRS-2002 Score ≥3	16.089 (3.291-78.65)	0.001	14.019 (2.514-78.166)	0.003

*Each variable in the multivariate regression analysis was adjusted for age and congestive heart failure (CHF).

**Table 7.** Regression Analysis Examining Factors Linked to Prolonged Hospitalization

	Analysis of Univariate		Analysis of Multivariate*	
	OR (95% GA)	Significance	OR (95% GA)	Significance
PANC-3 Score = 3	5.135 (1.928-13.676)	0.001	6.376 (2.298-17.687)	<0.001
NRS-2002 Score ≥3	9.764 (2.743-34.756)	<0.001	8.965 (2.415-33.273)	0.001
Age	1.032 (1.014-1.051)	<0.001	7.896 (3.441-18.117)	<0.001
Extrapancreatic fluid collection	2.818 (1.481-5.362)	0.002	3.529 (1.759-7.077)	<0.001
Peripancreatic fluid collection	2.32 (1.264-4.257)	0.007	2.697 (1.415-5.141)	0.003
Acute necrotizing collection	6.705 (2.137-21.034)	0.001	9.194 (2.763-30.594)	<0.001

*Each variable in the multivariate regression analysis was adjusted for age, hypertension (HT), and chronic kidney disease (CKD).
